# The antimicrobial possibilities of green tea

**DOI:** 10.3389/fmicb.2014.00434

**Published:** 2014-08-20

**Authors:** Wanda C. Reygaert

**Affiliations:** Department of Biomedical Sciences, Oakland University William Beaumont School of MedicineRochester, MI, USA

**Keywords:** antimicrobial, green tea, synergism, catechins, EGCG

## Abstract

Green tea is a popular drink, especially in Asian countries, although its popularity continues to spread across the globe. The health benefits of green tea, derived from the leaves of the *Camellia sinensis* plant, have been studied for many years. Fairly recently, researchers have begun to look at the possibility of using green tea in antimicrobial therapy, and the potential prevention of infections. The particular properties of catechins found in the tea have shown promise for having antimicrobial effects. There are four main catechins (polyphenols) found in green tea: (-)-epicatechin (EC), (-)-epicatechin-3-gallate (ECG), (-)-epigallocatechin (EGC), and (-)-epigallocatechin-3-gallate (EGCG). Three of these, ECG, EGC, and EGCG have been shown to have antimicrobial effects against a variety of organisms. These catechins have exhibited a variety of antimicrobial mechanisms. The results of studies on the antimicrobial effects of green tea have shown that the potential for preventive and therapeutic purposes is present. Further data collection on studies performed with human consumption during the course of infections, and studies on the occurrence of infections in populations that consume regular amounts of green tea will be necessary to complete the picture of its antimicrobial possibilities.

## Introduction

Tea is a very popular drink world-wide. It is produced from the plant *Camellia sinensis*, which is grown in at least 30 countries, and grows best in certain tropical and subtropical regions (Gupta et al., 2014). Tea is mainly produced as four varieties; white, green, Oolong, and black. White tea is made from very young tea leaves or buds; green tea is made from mature unfermented leaves; Oolong tea from partially fermented leaves; and black tea from fully fermented leaves (Jigisha et al., 2012; Gupta et al., 2014). Most of the black tea produced is consumed in the United States, Oolong tea is most popular in China and Taiwan, and green tea is most popular in China, Japan, and Korea (Cabrera et al., 2006). Among the health benefits that have been studied using green tea are: as an antioxidant, anti-inflammatory, anticarcinogenic, in cardiovascular health, oral health, and as an antimicrobial. Antioxidant effects come from the ability of green tea to limit the amount of free radicals by binding to reactive oxygen species (ROS) (Serafini et al., 2011; Jigisha et al., 2012; Gupta et al., 2014). Anti-inflammatory effects may be a result of increased production of IL-10, an anti-inflammatory cytokine. Inflammation is involved in, among other conditions, arthritis, cardiovascular disease, aging, and cancer (Serafini et al., 2011; Jigisha et al., 2012). The anticarcinogenic effects of green tea have been seen in many types of cancer, and the mechanisms may include inhibiting angiogenesis and cell growth, and inducing apoptosis in cancer cells (Serafini et al., 2011; Jigisha et al., 2012; Subramani and Natesh, 2013). Cardiovascular effects include the antioxidant and anti-inflammatory effects, and consumption of green tea has been shown to inhibit atherosclerosis, reduce lipid levels overall, and improve the ratio of LDL to HDL (Serafini et al., 2011; Jigisha et al., 2012). The effects for oral health are related to both teeth and gums. The main cause of dental caries is the bacteria *Streptococcus mutans*. Green tea has a direct **antimicrobial** effect on this bacteria, plus it seems to inhibit the attachment of the bacteria to oral surfaces. In addition, green tea is a natural source of fluoride (Jigisha et al., 2012; Gupta et al., 2014). Green tea has been shown to have antimicrobial effects against a variety of gram positive and gram negative bacteria (e.g., *Escherichia coli*, *Salmonella* spp., *Staphylococcus aureus*, *Enterococcus* spp.), some fungi (e.g., *Candida albicans*), and a variety of viruses (e.g., HIV, herpes simplex, influenza) (Jigisha et al., 2012; Steinmann et al., 2013). These antimicrobial effects will be discussed in more detail later in this paper.

KEY CONCEPT 1. Antimicrobial Antibacterial agents attack microorganisms through four main mechanisms: inhibiting cell wall synthesis (β-lactams drugs, such as the penicillins and cephalosporins), inhibiting protein synthesis (aminoglycosides, tetracyclines, etc.), inhibiting nucleic acid synthesis (fluoroquinolones), or inhibiting metabolic pathways (trimethoprim, sulfonamides). Antiviral agents may target host cell entry, interfere with various stages of viral replication or assembly, or interfere with virus release from the host cell. Since fungi are eukaryotes, antifungal agents have to key in on differences between human and fungal cell processes. Because the parasites are a widely diverse group of organisms, antiparasitic agents are quite often specific for a certain parasite or related parasite group (e.g., nematodes).

## Green tea composition

The medically important components of green tea are the polyphenols, most importantly the flavonoids. The main flavonoids in tea are the catechins, making up 30–40% of the water-soluble solids in green tea (Wang and Ho, 2009; Roowi et al., 2010). The different types of tea vary in the amount of catechins that they contain, with green tea containing the most, then Oolong tea, then black tea. The initial steaming process in the production of green tea destroys the enzyme polyphenol oxidase, thus protecting the polyphenol content. There are four main catechins in tea. (-)-epicatechin (EC), (-)-epicatechin-3-gallate (ECG), (-)-epigallocatechin (EGC), and (-)-epigallocatechin-3-gallate (EGCG). In green tea, EGCG is the most abundant, representing approximately 59% of the total catechins. EGC is next, making up approximately 19%. Then ECG, at 13.6%; and EC, at 6.4% (Cabrera et al., 2006; Jigisha et al., 2012). In addition to the type of tea, the amount of catechins can be affected by: where on the plant the leaves are harvested, leaf processing, geographical location, growing conditions, and tea preparation (Fernandez et al., 2002; Lin et al., 2003; Cabrera et al., 2006).

In order for any of the components of green tea to be a health benefit they have to have **bioavailability** This is most commonly assessed by measuring plasma, urine, and perhaps (in directed studies) tissue levels of the components after ingestion; usually by taking samples at regularly timed intervals. Various studies conducted using consumption of green tea beverage (Henning et al., 2004; Stalmach et al., 2009; Clifford et al., 2013), green tea extract (Lee et al., 1995; Yang et al., 1998; Henning et al., 2004), or specific catechins (Chow et al., 2001; Van Amelsvoort et al., 2001), have shown that ECG, EGCG, and metabolites of EC and EGC can be detected in blood plasma; and metabolites of EC and EGC only can be detected in urine. The peak concentration in blood plasma occurs at approximately 2 h after ingestion; and the peak concentration in urine occurs approximately 4–6 h after consumption. Some studies were performed using various dosages of the catechins, and show that the bioavailability of the catechins is directly proportional to the amount consumed (Nakagawa et al., 1997; Yang et al., 1998; Chow et al., 2001, 2003; Ullmann et al., 2003). Table 1 shows a summary of the results of these various bioavailability studies, giving data specifically for EGCG and EGC. The most abundant catechins in green tea, EGCG and EGC, also occur in the greatest concentration in the body after consumption (EGCG in plasma, and EGC in both plasma and urine).

**Table 1:** **Bioavailability of green tea catechins**.

**Catechin source**	**Initial dose**	**Plasma concentration (peak time) EGCG, EGC**	**EGC in urine/24 h (peak time)**	**References**
Green tea (bottled)	EGCG 230 μmol	EGCG ~55 *n*mol/L (~1.9 h)	~33 μmol	Clifford et al., 2013
	EGC 257 μmol	EGC ~205 *n*mol/L (~2.2 h)		
Green tea (brewed)	EGCG 214 mg	EGCG ~80 *n*mol/L (~1.3 h)	ND	Henning et al., 2004
	EGC 270 mg	EGC ~740 *n*mol/L (~1.3 h)		
Green tea extracts	EGCG 193 mg	EGCG ~150 *n*mol/L (~2.7 h)	ND	Henning et al., 2004
	EGC 25 mg	EGC 110 *n*mol/L (~2.6 h)		
Green tea extracts	EGCG 88 mg	EGCG ~135 ng/ml at 4 h	~3.0 mg (3–6 h)	Lee et al., 1995
	EGC 82 mg	EGC ~ 140 ng/ml at 1 h		
Green tea (bottled)	EGCG 235 μmol	EGCG ~55 nM (~1.9 h)	~33 μmol	Stalmach et al., 2009
	EGC 260 μmol	EGC ~126 nM (~2.2 h)		
EGCG EGC	EGCG 688 mg	EGCG ~1.3 μmol (~3.9 h)	ND	Van Amelsvoort et al., 2001
	EGC 459 mg	EGC ~5.0 μmol (~1.7 h)		
Green tea extracts	EGCG 110 mg	EGCG ~119 ng/ml (~1.6 h)	~3000 μg (4–6 h)	Yang et al., 1998
	EGC 102 mg	EGC ~148 ng/ml (~1.4 h)

KEY CONCEPT 2. Bioavailability Bioavailability is a measure of how much of a substance can be found in blood, tissues, urine, etc., after being introduced into the body. Enough of the substance must be present at the site of an infection in order to be useful in fighting the microorganism(s) involved.

In a previous study (Reygaert and Jusufi, 2013), it was projected that using a brewed preparation of Japanese green tea (containing approximately 20 mg of EGC per gram of tea) would result in approximately 150 mg of ECG per cup of tea. The amount of EGC excreted in the urine would be approximately 3.5 mg per cup of tea.

## Antimicrobial properties of green tea

In addition to the direct antimicrobial effects of catechins discussed below (damage to the bacterial cell membrane, inhibition of fatty acid synthesis, inhibition of enzyme activity, etc.), there are also some effects that may contribute to the total antimicrobial effect in infected individuals. These effects include inhibition of inflammation (particularly inflammation caused by oxidative stress, such as vascular), more specifically, by increasing the synthesis of nitric oxide (Yamakuchi et al., 2008), inhibiting angiotensin II and IL-6-induced C-reactive protein expression (Li et al., 2012), suppression of IL-6 and RANKL production in infected osteoblast-like cells (Ishida et al., 2007), inhibiting IL-8 production (Hirao et al., 2010), and inhibiting hyaluronidase activity (activated by chronic inflammation) by inhibition of IL-12 (Adcocks et al., 2002).

### Damage to the bacterial cell membrane

Many of the direct effects of tea catechins are a result of the catechins binding to the bacterial lipid bilayer cell membrane which then causes damage to the membrane (Sirk et al., 2008, 2009). This damage can then lead to a variety of related antimicrobial effects. Researchers studying *Escherichia coli* found that when exposed to green tea polyphenols, the bacterial response was changed regulation of 17 individual genes. Nine genes were upregulated and eight were downregulated. One of the major outcomes of this change in regulation was damage to the bacterial cell membrane (Cho et al., 2007). Tea catechins have less effect on gram negative bacterial cell membranes due to the fact that the LPS outer membrane of gram negative bacteria is negatively charged (Ikigai et al., 1993). Bacterial cell membrane damage inhibits the ability of the bacteria to bind to host cells (Sharma et al., 2012), and inhibits the ability of the bacteria to bind to each other to form biofilms, which are significant in pathogenesis (Blanco et al., 2005). Bacterial membrane damage also results in an inability of the bacteria to be able to secrete toxins (Sugita-Konishi et al., 1999; Shah et al., 2008). Directly related to the homeostasis of the bacterial cell membrane is fatty acid synthesis.

### Inhibition of fatty acid synthesis

Fatty acids in bacteria have important functions; as a component of phospholipid cell membranes (and mycolic acid in cell walls of mycobacteria), and as an excellent energy source. Researchers have recently begun to look at the potential of targeting fatty acid biosynthesis for antimicrobial drug development (Wang and Ma, 2013). Researchers have found that green tea components (especially EGCG) inhibit specific reductases (FabG, FabI) in bacterial type II fatty acid synthesis (Zhang and Rock, 2004; Li et al., 2006). Inhibition of fatty acid synthesis by green tea has also been found to inhibit bacterial production of toxic metabolites (Sakanaka and Okada, 2004).

### Inhibition of other enzyme activity

In addition to the enzymes involved in fatty acid synthesis, research with green tea has been shown to have effects on other essential bacterial enzymes. Researchers found that green tea catechins have an inhibitory effect on protein tyrosine phosphatase and cysteine proteinases in certain anaerobic oral bacteria (Okamoto et al., 2003, 2004). Researchers have also found that green tea catechins have the ability to interfere with DNA replication by interacting with, and thereby inhibiting the function of DNA gyrase (Grandišar et al., 2007). Studies on antifolate activity in microorganisms have shown that green tea polyphenols can inhibit the enzyme dihydrofolate reductase in bacteria and yeast, effectively blocking the ability of the microorganisms to synthesize folate (Navarro-Martinez et al., 2005, 2006). More recently is has been discovered that bioflavonoids (including those from green tea) could inhibit the activity of bacterial ATP synthase, reducing the ability of the microorganisms to produce enough energy (Chinnam et al., 2010).

### Other inhibitory effects

Researchers have found that green tea catechins have inhibitory effects on many other bacterial functions. One such effect is inhibiting synthesis of PBP2′ in methicillin-**resistant** *Staphylococcus aureus* **(MRSA)** This inhibition leads to reversal of resistance to β-lactam drugs (Yam et al., 1998). Another effect on bacteria is the inhibition by green tea catechins on the conjugative transfer of the R plasmid in *Escherichia coli* (Zhao et al., 2001a). This could lead to decreased sharing of antimicrobial genes between bacteria. Research on staphylococci has shown the ability of green tea catechins to inhibit the activity of efflux pumps. In this particular study the efflux pump was Tet(K) which is involved in resistance to tetracycline (Sudano Roccaro et al., 2004). This same type of activity could very well-occur with other efflux pumps, which could enhance the ability of certain drugs to have an antimicrobial effect. A study performed with *Helicobacter pylori* found that green tea catechins blocked the ability of the bacteria to bind to the toll-like receptor-4 (TLR-4) on gastric epithelial cells (Lee et al., 2004). This would lead to a drastic reduction in that organism's ability to cause gastric disease. There are also inhibitory effects found with viruses. Research with HIV-1 has shown that green tea EGCG binds to the CD4 T-cell receptor, blocking the binding of the virus. This ability to block viral cell binding has been proposed for use in therapy for HIV-1 infection (Williamson et al., 2006).

KEY CONCEPT 3. Resistant Microorganisms have a variety of methods that they use to overcome antimicrobial agents (antimicrobial-resistance). There are four main mechanisms that are used: inhibiting uptake of a drug, increased excretion of a drug (e.g., efflux pumps), structural or functional modification of the microorganism drug target, or inactivating the drug directly. This antimicrobial-resistance may be acquired from another similar organism, or a result of a mutation in one or more of the microorganism's own genes.

KEY CONCEPT 4. MRSA Methicillin-resistant *Staphylococcus aureus* (MRSA) have acquired a gene that allows production of an altered penicillin-binding protein (PBP), PBP2′. PBPs are transpeptidases that are used in the synthesis of the peptidoglycan layer of the bacterial cell wall. PBP2′ has a greatly reduced affinity for most of the β-lactam drugs, making these drugs ineffective against MRSA. This makes the treatment of infections with MRSA much more difficult and costly.

### Synergy of green tea with antimicrobials

Researchers have taken the concept of green tea catechins as having antimicrobial effects and expanded on this by performing studies that would determine if the green tea catechins might work synergistically with known antimicrobial agents. Many of these studies have focused on *Staphylococcus aureus* (particularly MRSA), *Staphylococcus epidermidis*, and *Escherichia coli*, among others. Examples of those studies have found that green tea catechins work synergistically with tetracycline against *S. aureus* and *S. epidermidis* (Sudano Roccaro et al., 2004; Fanaki et al., 2008); with penicillin against *S. epidermidis* (Haghjoo et al., 2013); with penicillin, oxacillin, ampicillin/sulbactam, and imipenem against MRSA (Hu et al., 2001, 2002; Zhao et al., 2001b; Stapleton et al., 2006; Aboulmagd et al., 2011); and with chloramphenicol, ciprofloxacin, and cefotaxime against isolates of *E. coli* having various levels of resistance, including extended-spectrum beta-lactamase **(ESBL)** producers (Fanaki et al., 2008; Cui et al., 2012a; Passat, 2012).

KEY CONCEPT 5. ESBL Extended-spectrum β-lactamase (ESBL) producing organisms are most often members of the gram negative *Enterobacteriaceae* family. Beta-lactamases are enzymes produced by the organism that are able to hydrolyze β-lactam drugs, rendering these drugs inactive. Like MRSA, the ESBLs have an increased resistance to penicillins and cephalosporins, but are sensitive to carbapenems and β-lactamase inhibitors (drugs that inhibit the activity of β-lactamases and are used together with a β-lactam drug, such as ampicillin/sulbactam). Many ESBL producers are also multidrug-resistant organisms (MRSOs), making treatment of these infections very difficult.

## Green tea catechins antimicrobial scope

A large variety of research has been performed assessing whether or not green tea has antimicrobial activities. A summary of examples of this research is included in Table 2. The research results shown do suggest that green tea may be effective against many organisms. The research is pretty well-balanced between gram positive and gram negative organisms, with a few viruses, fungi and a parasite also represented. The main gram positive organism represented is *S. aureus*. That is not surprising considering the ongoing menace of MRSA. The most commonly studied gram negative organism is *E. coli*. These results represent potentially useful information for battling the emerging threat of ESBL and Carbapenem-resistant *Enterobacteriaceae* **(CRE)** organisms. In a previous study, green tea extract was shown to be effective against *E. coli* strains isolated from UTIs. Many of these isolates (59/80) were multi-drug resistant (MDROs), and six of the strains were ESBLs (Reygaert and Jusufi, 2013). The results shown in Table 2, along with the possible synergistic activities mentioned earlier, suggest that green tea could be extremely useful as an antimicrobial agent. There are certain things to keep in mind when viewing the results in Table 2. First, is the type of green tea and the preparation used. The research may have used green tea from various locations around the globe. For the experiments, the preparation may have included freshly brewed green tea, green tea extracts (water, alcohol), or purified extracts of certain of the green tea catechins. There are also various other factors that may affect the composition of the tea (refer to the section on green tea composition). The second thing to pay attention to is the type of result reported. There are four basic types of results shown: growth in colony-forming units (CFUs), or plaque-forming units (PFUs) for viruses; minimal inhibitory concentration (MIC); the size of zones of inhibition; and the optical density (O.D.) of growth in liquid cultures. The percentage of increase or decrease of any of these parameters may also be shown. In addition, for viruses, other growth or detection measures made be shown. Overall, the results indicate that green tea has great potential as an antimicrobial agent.

**Table 2:** **Antimicrobial activity of green tea**.

**Source**	**Organisms studied**	**Antimicrobial activity**	**References**
Water extraction	MRSA	MIC as low as 0.78 mg/ml	Aboulmagd et al., 2011
Methanol extraction	*Enterococcus faecalis*	MIC 15.63 μg/ml	Aman et al., 2013
Water/ethyl acetate extraction	*Actinomyces*	MIC 6.25 mg/ml	Araghizadeh et al., 2013
	*actinomycetemcomitans*		
	*Porphyromonas gingivalis*	MIC 12.5 mg/ml	
	*Prevotella intermedia*	MIC 12.5 mg/ml	
	*Streptococcus mutans*	MIC 2.58-3.98 mg/ml	
Methanol extraction	*Escherichia coli*	MIC 0.8 mg/ml	Archana and Abraham, 2011
	*E. faecalis*	MIC 1.6 mg/ml	
	*Salmonella typhi*	MIC 1.2 mg/ml	
	*Staphylococcus aureus*	MIC 0.8 mg/ml	
Alcohol extraction	*E. coli*	MIC most ≤20 μg/ml	Bandyopadhyay et al., 2005
	*S. aureus*	MIC most ≤20 μg/ml	
	*Vibrio cholerae*	MIC most ≤30 μg/ml	
Standardized water extract	MRSA	MICs of 30 strains 50–180 μg/ml	Cho et al., 2008
Pure EGCG	*E. coli*	MIC 500 mg/ml	Cui et al., 2012b
	*Pseudomonas aeruginosa*	MIC 500 mg/ml	
	*S. aureus*	MIC 100 mg/ml	
	*S. mutans*	MIC 100 mg/ml	
Pure EGCG (Vero cells)	HSV-1	12× ↓ PFUs 50 μM	de Oliveira et al., 2013
Water extraction	*E. coli*	3× ↓ CFUs 10 mg/ml	Fanaki et al., 2008
Pure EGCG 50 μM	HIV-1 (infected lymphocytes)	No p24 core antigen detected	Fassina et al., 2002
Pure EGCG 10 μM	Hepatitis C virus (Huh 7.5.1 cells)	CPE reduced by 90%	Fukazawa et al., 2012
Pure EGCG	*Stenotrophomonas maltophilia* (40 isolates)	MIC ≤256 mg/ml	Gordon and Wareham, 2010
Pure EGCG	*Candida albicans*	MIC of 90% ≤250 mg/L	Hirasawa and Takada, 2004
Catechin mixture	*P. gingivalis*	MIC 1.0 mg/ml	Hirasawa et al., 2002
Water extraction 50 mg/ml	*Proteus mirabilis*	Zone 21.8 mm	Kim et al., 2008
	*Streptococcus pyogenes*	Zone 17.3 mm	
Pure EGCG 50 mg/ml	*Proteus mirabilis*	Zone 24.2 mm	Kim et al., 2008
	*Streptococcus pyogenes*	Zone 19.0 mm	
Pure EGCG 200 μM	*Listeria monocytogenes*	Decreased intracellular growth 10^6^ → 10^2^ CFUs	Kohda et al., 2008
Water extraction (in watermelon juice) to 0 CFUs	*E. coli*	250 mg/ml at 6 days	Kristanti and Punbusayakul, 2009
	*L. monocytogenes*	100 mg/ml at 4 days	
	*S. aureus*	75 mg/ml at 3 days	
	*Salmonella typhimurium*	175 mg/ml at 5 days	
Ethanol extract 4.5 mg	*E. coli*	Zone 13 mm	Kumar et al., 2012
	*P. aeruginosa*	Zone 10 mm	
	*S. aureus*	Zone 12 mm	
Ground tea leaves 1% wt/vol	*Bacillus cereus*	24% ↓ CFUs at 24 h	Lee et al., 2009
Water extraction	*L. monocytogenes*	MIC 0.68 mg/ml	Mbata et al., 2008
Methanol extraction		MIC 0.26 mg/ml	
Methanol extraction	*S. mutans*	MIC 100 mg/ml	Naderi et al., 2011
Pure EGCG	*Acinetobacter baumannii*	50%MIC 0.312 mg/ml	Osterburg et al., 2009
		90%MIC 0.625 mg/ml	
Water extraction 2 mg/ml	*Trypanosoma cruzi* (in mouse blood)	905 of organisms lysed	Paveto et al., 2004
Water extraction	MRSA	MIC 400 μg/ml	Radji et al., 2013
	MDR *P. aeruginosa*	MIC 800 μg/ml	
Water extraction	*Aspergillus niger*	MIC 50 mg/ml	Rao et al., 2014
	*P. aeruginosa*	MIC 50 mg/ml	
	*S. aureus*	MIC 50 mg/ml	
Standardized water extract	*E. coli* (79 UPEC strains)	MICs ≤2.5–4.0 mg/ml	Reygaert and Jusufi, 2013
Water extraction	Influenza viruses H1N1	0 PFUs at 500 μg/ml	Shin et al., 2012
Ethanol extraction	H3N2 (all in MDCK cells)	0 PFUs at 500 μg/ml	
Pure EGCG	H5N2	0 PFUs at 500 μg/ml	
Pure EGCG 12.5 μg/ml	MRSA	Oxacillin MIC reduced to 1 μg/ml	Stapleton et al., 2006
Water extraction 50 μg/ml	Influenza viruses H1N1	PFUs ↓ 100%	Song et al., 2005
	H3N2 (all in MDCK cells)	PFUs ↓ 90%	
	Influenza B	PFUs ↓ 95%	
Ethanol extract 9.375 mg	*Helicobacter pylori*	Zone 7.5 mm	Stoicov et al., 2009
Standardized green tea extract 40 μg/ml	Hepatitis B virus (in HepG2-N10 cells)	99% ↓ HBsAg	Xu et al., 2008
		93% ↓ HBeAg	
Water extraction	*S. aureus*	MIC 0.28 mg/ml	Yam et al., 1997
	*S. epidermidis*	MIC 0.41 mg/ml	
	*Yersinia enterocolitica*	MIC 0.41 mg/ml	
Pure EGCG 100 μg/ml	MRSA	O.D. ↓ 0	Zhao et al., 2001b
	MSSA	O.D. ↓ 0	
	*S. epidermidis*	O.D. ↓ 0

KEY CONCEPT 6. CRE Carbapenem-resistant *Enterobacteriaceae* (CREs) are similar to ESBLs. These organisms are resistant to all β-lactam drugs, and are not inactivated by β-lactamase inhibitors. The most common ESBL-producing organisms are *Escherichia coli* and *Klebsiella pneumoniae*. Treatment options are extremely limited for infections involving these organisms.

## Discussion

The data discussed shows that green tea catechins EGCG and EGC are bioavailable in plasma (both) and urine (EGC only) at potentially therapeutic levels and in a timely manner (see Table 1). Antimicrobial studies using various green tea extracts have shown that there is a huge potential for the use of green tea in antimicrobial therapy (see Table 2). In a previous study, the amount of EGC from brewed Japanese green tea excreted in the urine was calculated to be approximately 3.5 mg per cup of tea. Since the results of the study showed that most of the bacterial strains tested were susceptible to EGC levels of ≤0.72 mg, one cup of tea should be able to control growth of the bacteria for up to 6 h or even longer (Reygaert and Jusufi, 2013).

While the research results that have been discussed suggest that green tea may be effective against many organisms, there are certain issues that need to be addressed concerning these results. The first issue is how the results are displayed. The difficulty of assessing these types of results, and how these results relate to each other among the different research groups, can be confusing. This brings us to the second major issue. Various agencies, such as the Clinical and Laboratories Standards Institute (CLSI) in the United States, have strict protocols for the determination of antimicrobial susceptibility. Most of the research results that have been performed using green tea have not necessarily followed any of these types of guidelines. Therefore, the results of these experiments may differ from what the results would have been if strict protocols had been used. In the future, it will be most helpful if researchers would take the time to use these protocols. That will make comparing the results, and assessing if the results are meaningful so much easier. In addition (and most importantly), in order for the results to be able to be translated into clinical use, the protocols will need to be followed. That having been addressed, it is still very encouraging to look at the research performed and see that green tea could very possibly be an effective antimicrobial agent, especially against multidrug-resistant strains, and in particular, MRSA and ESBL producing organisms. Hopefully, in the future, researchers will be able to study the effects of green tea on infections in humans. This type of research is a critical part of determining the antimicrobial capabilities of green tea. It might possibly be incorporated into research with other antimicrobial compounds. Perhaps naturopathic practitioners could begin to collect data on patients using green tea. With emerging multidrug-resistant organisms and the lack of effective new antimicrobial drugs being produced, we cannot afford to ignore the potential of green tea.

### Conflict of interest statement

The author declares that the research was conducted in the absence of any commercial or financial relationships that could be construed as a potential conflict of interest.

## References

[B1] Aboulmagd E. Al-Mohammed H. I. Al-Badry S. (2011). Synergism and postantibiotic effect of green tea extract and imipenem against methicillin-resistant *Staphylococcus aureus*. Microbiol. J. 1, 89–96 10.3923/mj.2011.89.96

[B2] Adcocks C. Collin P. Buttle D. J. (2002). Catechins from green tea (*Camellia sinensis*) inhibitbovine and human cartilage proteoglycan and type II collagen degradation *in vitro*. J. Nutr. 132, 341–346 11880552 10.1093/jn/132.3.341

[B3] Aman S. Naim A. Siddiqi R. Naz S. (2013). Antimicrobial polyphenols from small tropicalfruits, tea and spice oilseeds. Food Sci. Technol. Int. 20, 241–251 10.1177/1082013213482476 23703103

[B4] Araghizadeh A. Kohanteb J. Fani M. M. (2013). Inhibitory activity of green tea (*Camellia sinensis*) extract on some clinically isolated cariogenic and periodontopathic bacteria. Med. Princ. Pract. 22, 368–372 10.1159/000348299 23485656 PMC5586764

[B5] Archana S. Abraham J. (2011). Comparative analysis of antimicrobial activity of leaf extracts from fresh green tea, commercial green tea and black tea on pathogens. J. Appl. Pharm. Sci. 1, 149–152

[B6] Bandyopadhyay D. Chatterjee T. K. Dasgupta A. Lourduraja J. Dastidar S. G. (2005). *In vitro* and *in vivo* antimicrobial action of tea: the commonest beverage of Asia. Biol. Pharm. Bull. 28, 2125–2127 10.1248/bpb.28.2125 16272702

[B7] Blanco A. R. Sudano-Roccaro A. Spoto G. C. Nostro A. Rusciano D. (2005). Epigallocatechin gallate inhibits biofilm formation by ocular staphylococcal isolates. Antimicrob. Agents Chemother. 49, 4339–4343 10.1128/AAC.49.10.4339-4343.2005 16189116 PMC1251539

[B8] Cabrera C. Artacho R. Gimenez R. (2006). Beneficial effects of green tea – a review. J. Am. Coll. Nutr. 25, 79–99 10.1080/07315724.2006.10719518 16582024

[B9] Chinnam N. Dadi P. K. Sabri S. A. Ahmad M. Kabir M. A. Ahmad Z. (2010). Dietary bioflavonoids inhibit *Escherichia coli* ATP synthesis in a differential manner. Int. J. Biol. Macromol. 46, 478–486 10.1016/j.ijbiomac.2010.03.009 20346967 PMC2862773

[B10] Cho Y. S. Schiller N. L. Kahng H. Y. Oh K. H. (2007). Cellular responses and proteomic analysis of *Escherichia coli* exposed to green tea polyphenols. Curr. Microbiol. 55, 501–506 10.1007/s00284-007-9021-8 17874165

[B11] Cho Y. S. Schiller N. L. Oh K. H. (2008). Antibacterial effects of green tea polyphenols on clinical isolates of methicillin-resistant *Staphylococcus aureus*. Curr. Microbiol.. 57, 542–546 10.1007/s00284-008-9239-0 18781360

[B12] Chow H. H. Cai Y. Alberts D. S. Hakim I. Dorr R. Shahi F. (2001). Phase I pharmacokinetic study of tea polyphenols following single-dose administration of epigallocatechin gallate and polyphenon E. Cancer Epidemiol. Biomarkers Prev. 10, 53–58 11205489

[B13] Chow H. H. Cai Y. Hakim I. A. Crowell J. A. Shahi F. Brooks C. A. (2003). Pharmacokinetics and safety of green tea polyphenols after multiple- dose administration of epigallocatechin gallate and polyphenon E in healthy individuals. Clin. Cancer Res. 9, 3312–3319 12960117

[B14] Clifford M. N. van der Hooft J. J. Crozier A. (2013). Human studies on the absorption, distribution, metabolism, and excretion of tea polyphenols. Am. J. Clin. Nutr. 98, 1619S–1630S 10.3945/ajcn.113.058958 24172307

[B15] Cui Y. Kim S. H. Kim H. Yeom J. Ko K. Park W. (2012a). AFM probing the mechanism of synergistic effects of the green tea polyphenol (-)-epigallocatechin-3-gallate (EGCG) with cefotaxime against extended-spectrum beta-lactamase (ESBL)-producing *Escherichia coli*. PLoS ONE 7:e48880 10.1371/journal.pone.0048880 23152812 PMC3496731

[B16] Cui Y. Oh Y. J. Youn M. Lee I. Pak H. K. Park W. (2012b). AFM study of the differential inhibitory effects of the green tea polyphenol (-)-epigallocatechin-3-gallate (EGCG) against gram-positive and gram-negative bacteria. Food Microbiol. 29, 80–87 10.1016/j.fm.2011.08.019 22029921

[B17] de Oliveira A. Adams A. D. Lee L. H. Murray S. R. Hsu S. D. Hammond J. R. (2013). Inhibition of herpes simplex virus type 1 with the modified green tea polyphenol palmitoyl-epigallocatechin gallate. Food Chem. Toxicol. 52, 207–215 10.1016/j.fct.2012.11.006 23182741 PMC3703635

[B18] Fanaki N. H. Kassem M. A. Fawzi M. A. Dabbous F. (2008). Influence of aqueous green tea extract on the antimicrobial activity of some antibiotics against multiresistant clinical isolates. Egypt. J. Med. Microbiol. 17, 449–460

[B19] Fassina G. Buffa A. Benelli R. Varnier O. E. Noonan D. M. Albini A. (2002). Polyphenolic antioxidant (-)-epigallocatechin-3-gallate from green tea as a candidate anti-HIV agent. AIDS 16, 939–941 10.1097/00002030-200204120-00020 11919502

[B20] Fernandez P. L. Pablos F. Martin M. J. Gonzalez A. G. (2002). Study of catechin and xanthine tea profiles as geographical tracers. J. Agric. Food Chem. 59, 1833–1839 10.1021/jf0114435 11902920

[B21] Fukazawa H. Suzuki T. Wakita T. Murakami Y. (2012). A cell-based microplate colorimetric screen identifies 7,8-benzoflavone and green tea gallate catechins a inhibitors of the hepatitis C virus. Biol. Pharm. Bull. 35, 1320–1327 10.1248/bpb.b12-00251 22863932

[B22] Gordon N. C. Wareham D. W. (2010). Antimicrobial activity of the green tea polyphenol (-)-epigallocatechin-3-gallate (EGCG) against clinical isolates of *Stenotrophomonas maltophilia*. Int. J. Antimicrob. Agents 36, 129–131 10.1016/j.ijantimicag.2010.03.025 20472404

[B23] Grandišar H. Pristovšek P. Plaper A. Jerala R. (2007). Green tea inhibit bacterial DNA gyrase by interaction with its ATP binding site. J. Med. Chem. 50, 264–271 10.1021/jm060817o 17228868

[B24] Gupta D. A. Bhaskar D. J. Gupta R. K. Karim B. Jain A. Dalai D. R. (2014). Green tea: a review on its natural anti-oxidant therapy and cariostatic benefits. Biol. Sci. Pharm. Res. 2, 8–12 Available online at: http://journalissues.org/abstract/gupta-et-al/

[B25] Haghjoo B. Lee L. H. Habiba U. Tahir H. Olabi M. Chu T. C. (2013). The synergistic effects of green tea polyphenols and antibiotics against potential pathogens. Adv. Biosci. Biotechnol. 4, 959–967 10.4236/abb.2013.411127

[B26] Henning S. M. Niu Y. Lee N. H. Thames G. D. Minutti R. R. Wang H. (2004). Bioavailability and antioxidant activity of tea flavanols after consumption of green tea, black tea, or a green tea extract supplement. Am. J. Clin. Nutr. 80, 1558–1564 15585768 10.1093/ajcn/80.6.1558

[B27] Hirao K. Yumoto H. Nakanishi T. Mukai K. Takahashi K. Takegawa D. (2010). Tea catechins reduce inflammatory reactions via mitogen-activated protein kinase pathways in toll-like receptor 2 ligand-stimulated dental pulp cells. Life Sci. 86, 654–660 10.1016/j.lfs.2010.02.017 20176036

[B28] Hirasawa M. Takada K. (2004). Multiple effects of green tea catechin on the antifungal activity of antimycotics against *Candida albicans*. J. Antimicrob. Chemother. 53, 225–229 10.1093/jac/dkh046 14688042

[B29] Hirasawa M. Takada K. Makimura M. Otake S. (2002). Improvement of periodontal status by green tea catechin using a local delivery system: a clinical pilot study. J. Periodontal Res. 37, 433–438 10.1034/j.1600-0765.2002.01640.x 12472837

[B30] Hu Z. Q. Zhao W. H. Asano N. Yoda Y. Hara Y. Shimamura T. (2002). Epigallocatechin gallate synergistically enhances the activity of carbapenems against methicillin-resistant *Staphylococcus aureus*. Antimicrob. Agents Chemother. 46, 558–560 10.1128/AAC.46.2.558-560.2002 11796378 PMC127028

[B31] Hu Z. Q. Zhao W. H. Hara Y. Shimamura T. (2001). Epigallocatechin gallate synergy with ampicillin/sulbactam against 28 clinical isolates of methicillin-resistant *Staphylococcus aureus*. J. Antimicrob. Chemother. 48, 361–364 10.1093/jac/48.3.361 11533000

[B32] Ikigai H. Nakae T. Hara Y. Shimamura T. (1993). Bactericidal catechins damage the lipid bilayer. Biochim. Biophys. Acta 1147, 132–136 10.1016/0005-2736(93)90323-R 8466924

[B33] Ishida I. Kohda C. Yanagawa Y. Miyaoka H. Shimamura T. (2007). Epigallocatechin gallate suppresses expression of receptor activator of NF-kappaB ligand (RANKL) in *Staphylococcus aureus* infection in osteoblast-like NRG cells. J. Med. Microbiol. 56, 1042–1046 10.1099/jmm.0.47029-0 17644710

[B34] Jigisha A. Nishant R. Navin K. Pankaj G. (2012). Green tea: a magical herb with miraculous outcomes. Int. Res. J. Pharm. 3, 139–148 Available online at: http://www.irjponline.com/admin/php/uploads/1083_pdf.pdf

[B35] Kim S. H. Lee L. S. Bae S. M. Han S. J. Lee B. R. Ahn W. S. (2008). Antimicrobial and antifungal effects of a green tea extract against vaginal pathogens. J. Womens Med. 1, 27–36 Available online at: http://210.101.116.28/W_files/kiss8/40323392_pv.pdf

[B36] Kohda C. Yanagawa Y. Shimamura T. (2008). Epigallocatechin gallate inhibits intracellular survival of *Listeria monocytogenes* in macrophages. Biochem. Biophys. Res. Commun. 365, 310–315 10.1016/j.bbrc.2007.10.190 17996193

[B37] Kristanti R. A. Punbusayakul N. (2009). Inhibitory effect of commercial Assam green tea infusion in watermelon juice. Asian J. Food Agroind. 2, S249–S255

[B38] Kumar A. Kumar A. Thakur P. Patil S. Payal C. Kumar A. (2012). Antibacterial activity of green tea (*Camellia sinensis*) extracts against various bacteria isolated from environmental sources. Recent Res. Sci. Technol. 4, 19–23 Available online at: http://recent-science.com/index.php/rrst/article/view/10583/5370

[B39] Lee K. M. Yeo M. Choue J. S. Jin J. H. Park S. J. Cheong J. Y. (2004). Protective mechanism of epigallocatechin-3-gallate against *Helicobacter pylori*-induced gastric epithelial cytotoxicity via the blockage of TLR-4 signaling. Helicobacter 9, 632–642 10.1111/j.1083-4389.2004.00281.x 15610077

[B40] Lee M. J. Wang Z. Y. Li H. Chen L. Sun Y. Gobbo S. (1995). Analysis of plasma and urinary tea polyphenols in human subjects. Cancer Epidemiol. Biomarkers Prev. 4, 393–399 7655336

[B41] Lee S. Y. Gwon S. Y. Kim S. J. Moon B. K. (2009). Inhibitory effect of commercial green tea and rosemary leaf powders on the growth of foodborne pathogens in laboratory media and oriental-style rice cakes. J. Food Prot. 72, 1107–1111 19517743 10.4315/0362-028x-72.5.1107

[B42] Li B. H. Zhang R. Du Y. T. Sun Y. H. Tian W. X. (2006). Inactivation mechanism of the beta-ketoacyl-[acyl carrier protein] reductase of bacterial type-II fatty acid synthesis by epigallocatechin gallate. Biochem. Cell Biol. 84, 755–762 10.1139/o06-047 17167539

[B43] Li M. Liu J. T. Pang X. M. Han C. J. Mao J. J. (2012). Epigallocatechin-3-gallate inhibits angiotensin II and interleukin-6-induced C-reactive protein production in macrophages. Pharmacol. Rep. 64, 912–918 10.1016/S1734-1140(12)70886-1 23087143

[B44] Lin Y. S. Tsai Y. J. Tsay J. S. Lin J. K. (2003). Factors affecting the levels of tea polyphenols and caffeine in tea leaves. J Agric. Food Chem. 51, 1864–1873 10.1021/jf021066b 12643643

[B45] Mbata T. I. Debiao L. U. Saikia A. (2008). Antibacterial activity of the crude extract of Chinese green tea (*Camellia sinensis*) on *Listeria monocytogenes*. Afr. J. Biotechnol. 7, 1571–1573 10.5897/AJB06.316

[B46] Naderi N. J. Naikan M. Kharazi Fard M. J. Zardi S. (2011). Antibacterial activity of Iranian green and black tea on *Streptococcus mutans*: an *in vitro* study. J. Dent. (Tehran) 8, 55–59 21998809 PMC3184736

[B47] Nakagawa K. Okuda S. Miyazawa T. (1997). Dose-dependent incorporation of tea catechins, (-)-epigallocatechin-3-gallate and (-)-epigallocatechin, into human plasma. Biosci. Biotechnol. Biochem. 61, 1981–1985 10.1271/bbb.61.1981 9438978

[B48] Navarro-Martinez M. D. Garcia-Canovas F. Rodriguez-Lopez J. N. (2006). Tea polyphenol epigallocatechin-3-gallate inhibits ergosterol synthesis by disturbing folic acid metabolism in *Candida albicans*. J. Antimicrob. Chemother. 57, 1083–1092 10.1093/jac/dkl124 16585130

[B49] Navarro-Martinez M. D. Navarro-Peran E. Cabezas-Herrera J. Ruiz-Gomez J. Garcia-Canovas F. Rodriguez-Lopez J. N. (2005). Antifolate activity of epigallocatechin gallate against *Stenotrophomonas maltophilia*. Antimicrob. Agents Chemother. 49, 2914–2920 10.1128/AAC.49.7.2914-2920.2005 15980368 PMC1168674

[B50] Okamoto M. Leumg K. P. Ansai T. Sugimoto A. Maeda N. (2003). Inhibitory effects of green tea catechins on protein tyrosine phosphatase in *Prevotella intermedia*. Oral Microbiol. Immunol. 18, 192–195 10.1034/j.1399-302X.2003.00056.x 12753472

[B51] Okamoto M. Sugimoto A. Leung K. P. Nakayama K. Kamaguchi A. Maeda N. (2004). Inhibitory effect of green tea catechins on cysteine proteinases in *Porphyromonas gingivalis*. Oral Microbiol. Immunol. 19, 118–120 10.1046/j.0902-0055.2003.00112.x 14871352

[B52] Osterburg A. Gardner J. Hyon S. H. Neely A. Babcock G. (2009). Highly antibiotic-resistant *Acinetobacter baumannii* clinical isolates are killed by the green tea polyphenol (-)-epigallocatechin-3-gallate (EGCG). Clin. Microbiol. Infect. 15, 341–346 10.1111/j.1469-0691.2009.02710.x 19431221

[B53] Passat D. N. (2012). Interactions of black and green tea water extracts with antibiotics activity in local urinary isolated *Escherichia coli*. J. AlNahrain Univ. 15, 134–142 Available online at: http://www.jnus.org/pdf/1/2012/1/636.pdf

[B54] Paveto C. Guida M. C. Esteva M. I. Martino V. Coussio J. Flawia M. M. (2004). Anti-Trypanosoma cruzi activity of green tea (*Camellia sinensis*) catechins. Antimcrob. Agents Chemother. 48, 69–74 10.1128/AAC.48.1.69-74.2004 14693520 PMC310206

[B55] Radji M. Agustama R. A. Elya B. Tjampkasari C. R. (2013). Antimicrobial activity of green tea extract against isolates of methicillin-resistant *Staphylococcus aureus* and multi-drug resistant *Pseudomonas aeruginosa*. Asian Pac. J. Trop. Biomed. 3, 663–667 10.1016/S2221-1691(13)60133-1 23905026 PMC3703562

[B56] Rao S. Timsina B. Nadumane V. K. (2014). Antimicrobial effects of medicinal plants and their comparative cytotoxic effects on HEPG2 cell line. Int. J. Pharm. Pharm. Sci. 6, 101–105 Available online at: http://www.ijppsjournal.com/Vol6Issue1/7601.pdf 24577925

[B57] Reygaert W. Jusufi I. (2013). Green tea as an effective antimicrobial for urinary tract infections caused by *Escherichia coli*. Front. Microbiol. 4:162 10.3389/fmicb.2013.00162 23785367 PMC3684790

[B58] Roowi S. Stalmach A. Mullen W. Lean M. E. Edwards C. A. Crozier A. (2010). Green tea flavan-3-ols: colonic degradation and urinary excretion of catabolites by humans. J. Agric. Food Chem. 58, 1296–1304 10.1021/jf9032975 20041649

[B59] Sakanaka S. Okada Y. (2004). Inhibitory effects of green tea polyphenols on the production of a virulence factor of the periodontal-disease-causing anaerobic bacterium *Porphyromonas gingivalis*. J. Agric. Food Chem. 52, 1688–1692 10.1021/jf0302815 15030231

[B60] Serafini M. Del Rio D. Yao D. N. Bettuzzi S. Peluso I. (2011). Health benefits of tea, in Herbal Medicine: Biomolecular and Clinical Aspects, 2nd Edn, Chapter 12, eds Benzie I. F. F. Wachtel-Galor S. (Boca Rotan, FL: CRC Press), 239–262 10.1201/b10787-13

[B61] Shah S. Stapleton P. D. Taylor P. W. (2008). The polyphenol (-)-epicatechin gallate disrupts the secretion of virulence-related proteins by *Staphylococcus aureus*. Lett. Appl. Microbiol. 46, 181–185 10.1111/j.1472-765X.2007.02296.x 18069979 PMC2241664

[B62] Sharma A. Gupta S. Sarethy I. P. Dang S. Gabrani R. (2012). Green tea extract: possible mechanism and antibacterial activity on skin pathogens. Food Chem. 135, 672–675 10.1016/j.foodchem.2012.04.143 22868144

[B63] Shin W. J. Kim Y. K. Lee K. H. Seong B. L. (2012). Evaluation of the antiviral activity of a green tea solution as a hand-wash disinfectant. Biosci. Biotechnol. Biochem. 76, 581–584 10.1271/bbb.110764 22451404

[B64] Sirk T. W. Brown E. F. Friedman M. Sum A. K. (2009). Molecular binding of catechins to biomembranes: relationship to biological activity. J. Agric. Food Chem. 57, 6720–6728 10.1021/jf900951w 19572638

[B65] Sirk T. W. Brown E. F. Sum A. K. Friedman M. (2008). Molecular dynamics study on the biophysical interactions of seven green tea catechins with lipid bilayers of cell membranes. J. Agric. Food Chem. 56, 7750–7758 10.1021/jf8013298 18672886

[B66] Song J. M. Lee K. H. Seong B. L. (2005). Antiviral effect of catechins in green tea on influenza virus. Antiviral Res. 68, 66–74 10.1016/j.antiviral.2005.06.010 16137775

[B67] Stalmach A. Troufflard S. Serafini M. Crozier A. (2009). Absorption, metabolism and excretion of Choladi green tea flavan-3-ols by humans. Mol. Nutr. Food Res. 53, S44–S53 10.1002/mnfr.200800169 18979506

[B68] Stapleton P. D. Shah S. Hara Y. Taylor P. W. (2006). Potentiation of catechin gallate-mediated sensitization of *Staphylococcus aureus* to oxacillin by nongalloylated catechins. Antimicrob. Agents Chemother. 50, 752–755 10.1128/AAC.50.2.752-755.2006 16436737 PMC1366925

[B69] Steinmann J. Buer J. Pietschmann T. Steinmann E. (2013). Anti-infective properties of epigallocatechin-3-gallate (ECGC), a component of green tea. Br. J. Pharmacol. 168, 1059–1073 10.1111/bph.12009 23072320 PMC3594666

[B70] Stoicov C. Saffari R. Houghton J. M. (2009). Green tea inhibits *Helicobacter pylori* growth *in vivo* and *in vitro*. Int. J. Antimicrob. Agents 33, 473–478 10.1016/j.ijantimicag.2008.10.032 19157800 PMC2694061

[B71] Subramani C. Natesh R. K. (2013). Molecular mechanisms and biological implications of green tea polyphenol, (-)-epigallocatechin -3- gallate. Int. J. Pharm. Biosci. Tech. 1, 54–63

[B72] Sudano-Roccaro A. Blanco A. R. Giuliano F. Rusciano D. Enea V. (2004). Epigallocatechin-gallate enhances the activity of tetracycline in staphylococci by inhibiting its efflux from bacterial cells. Antimicrob. Agents Chemother. 48, 1968–1973 10.1128/AAC.48.6.1968-1973.2004 15155186 PMC415601

[B73] Sugita-Konishi Y. Hara-Kudo Y. Amano F. Okubo T. Aoi N. Iwaki M. (1999). Epigallocatechin gallate and gallocatechin gallate in green tea catechins inhibit extracellular release of vero toxin from enterohemorrhagic *Escherichia coli* O157:H7. Biochim. Biophys. Acta 1472, 42–50 10.1016/S0304-4165(99)00102-6 10572924

[B74] Ullmann U. Haller J. Decourt J. P. Girault N. Girault J. Richard-Caudron A. S. (2003). A single ascending dose study of epigallocatechin gallate in healthy volunteers. J. Int. Med. Res. 31, 88–101 10.1177/147323000303100205 12760312

[B75] Van Amelsvoort J. M. Van Hof K. H. Mathot J. N. Mulder T. P. Wiersma A. Tijburg L. B. (2001). Plasma concentrations of individual tea catechins after a single oral dose in humans. Xenobiotica 31, 891–901 10.1080/00498250110079149 11780763

[B76] Wang Y. Ho C. T. (2009). Polyphenolic chemistry of tea and coffee: a century of progress. J. Agric. Food Chem. 57, 8109–8114 10.1021/jf804025c 19719133

[B77] Wang Y. Ma S. (2013). Recent advances in inhibitors of bacterial fatty acid synthesis type II (FASII) system enzymes as potential antibacterial agents. ChemMedChem 8, 1589–1608 10.1002/cmdc.201300209 23894064

[B78] Williamson M. P. McCormick T. G. Nance C. L. Shearer W. T. (2006). Epigallocatechin gallate, the main polyphenol in green tea, binds to the T-cell receptor, CD4: potential for HIV- 1 therapy. J. Allergy Clin. Immunol. 118, 1369–1374 10.1016/j.jaci.2006.08.016 17157668

[B79] Xu J. Wang J. Deng F. Hu Z. Wang H. (2008). Green tea extract and its major component epigallocatechin gallate inhibits hepatitis B virus *in vitro*. Antiviral Res. 78, 242–249 10.1016/j.antiviral.2007.11.011 18313149

[B80] Yam T. S. Hamilton-Miller J. M. Shah S. (1998). The effect of a component of tea (*Camellia sinensis*) on methicillin resistance, PBP2' synthesis, and beta-lactamase production in *Staphylococcus aureus*. J. Antimicrob. Chemother. 42, 211–216 10.1093/jac/42.2.211 9738838

[B81] Yam T. S. Shah S. Hamilton-Miller J. M. (1997). Microbiological activity of whole and fractionated crude extracts of tea (*Camellia sinensis*), and of tea components. FEMS Microbiol. Lett. 152, 169–174 10.1111/j.1574-6968.1997.tb10424.x 9228784

[B82] Yamakuchi M. Bao C. Ferlito M. Lowenstein C. J. (2008). Epigallocatechin gallate inhibits endothelial exocytosis. Biol. Chem. 389, 935–941 10.1515/BC.2008.095 18627310 PMC2846414

[B83] Yang C. S. Chen L. Lee M. J. Balentine D. Kuo M. C. Schantz S. P. (1998). Blood and urine levels of tea catechins after ingestion of different amounts of green tea by human volunteers. Cancer Epidemiol. Biomarkers Prev. 7, 351–354 9568793

[B84] Zhang Y. M. Rock C. O. (2004). Evaluation of epigallocatechin gallate and related plant polyphenols as inhibitors of the FabG and FabI reductases of bacterial type II fatty-acid synthesis. J. Biol. Chem. 279, 30994–31001 10.1074/jbc.M403697200 15133034

[B85] Zhao W. H. Hu Z. Q. Hara Y. Shimamura T. (2001a). Inhibition by epigallocatechin gallate (EGCg) of conjugative R plasmid transfer in *Escherichia coli*. J. Infect. Chemother. 7, 195–197 10.1007/s101560100035 11810584

[B86] Zhao W. H. Hu Z. Q. Okubo S. Hara Y. Shimamura T. (2001b). Mechanism of synergy between epigallocatechin gallate and beta-lactams against methicillin-resistant *Staphylococcus aureus*. Antimicrob. Agents Chemother. 45, 1737–1742 10.1128/AAC.45.6.1737-1742.2001 11353619 PMC90539

